# Hydrolysis of Oligosaccharides by a Thermostable α-Galactosidase from *Termitomyces eurrhizus*

**DOI:** 10.3390/ijms161226159

**Published:** 2015-12-08

**Authors:** Weiwei Zhang, Fang Du, Li Wang, Liyan Zhao, Hexiang Wang, Tzi Bun Ng

**Affiliations:** 1 State Key Laboratory for Agrobiotechnology and Department of Microbiology, China Agricultural University, Beijing 100193, China; lczww2003@cau.edu.cn (W.Z.); haoyou0102@163.com (L.W.); 2 Shaanxi Microbiology Research Institute, No. 76 Xiying Road, Xi’an 710043, China; duf35@163.com; 3 College of Food Science and Technology, Nanjing Agricultural University, Weigang, Nanjing 210095, China; zhlychen@njau.edu.cn; 4 School of Biomedical Sciences, Faculty of Medicine, The Chinese University of Hong Kong, Shatin, New Territories, Hong Kong, China

**Keywords:** characterization, degradation, ITS, *Termitomyces eurrhizus*, TLC

## Abstract

The genus of *Termitomyces* purchased from the market has been identified as *Termitomyces eurrhizus* using the Internal Transcribed Spacer (ITS) method. An α-galactosidase from *T. eurrhizus* (TEG), a monomeric protein with a molecular mass of 72 kDa, was purified 146 fold by employing ion exchange chromatography and gel filtration. The optimum pH and temperature was 5.0 and 60 °C, respectively. TEG was stable over pH 2–6, and also exhibited good thermostablility, retaining 100% of the original activity after incubation at 60 °C for 2 h. Inhibition of the enzyme activity by *N*-bromosuccinimide (NBS) constituted evidence for an essential role of tryptophan in the catalytic action of the isolated enzyme. Besides 4-nitro-phenyl α-d-galactophyranoside (pNPGal), natural substrates could also be effectively hydrolyzed by TEG. Results of thin-layer chromatography (TLC) revealed complete enzymatic hydrolysis of raffinose and stachyose to galactose at 50 °C within 6 h. These properties of TEG advocate its utilization for elevating the nutritional value of soymilk.

## 1. Introduction

Different mushrooms of the genus *Termitomyces* like *Termitomyces striatus*, *T. eurrhizus*, *Termitomyces microcarpus*, *Termitomyces robustus* and *Termitomyces clypeatus*, which grow abundantly in termite nests, have been identified as wild edible mushrooms with high nutritive value. Several useful materials have been isolated from mushroom belonging to the genus *Termitomyces*. Water-soluble and water-insoluble polysaccharides have been isolated from *T. microcarpus* and *T. eurrhizus* and exhibit antitumour properties [1,2]. A hydrogen peroxide-dependent phenol oxidase essential for degradation of lignin was isolated from *Termitomyces albuminosus* [3]. An alkaline protease and a ribonuclease have also been isolated from *Termitomyces globules* [4,5].

**Figure 1:**
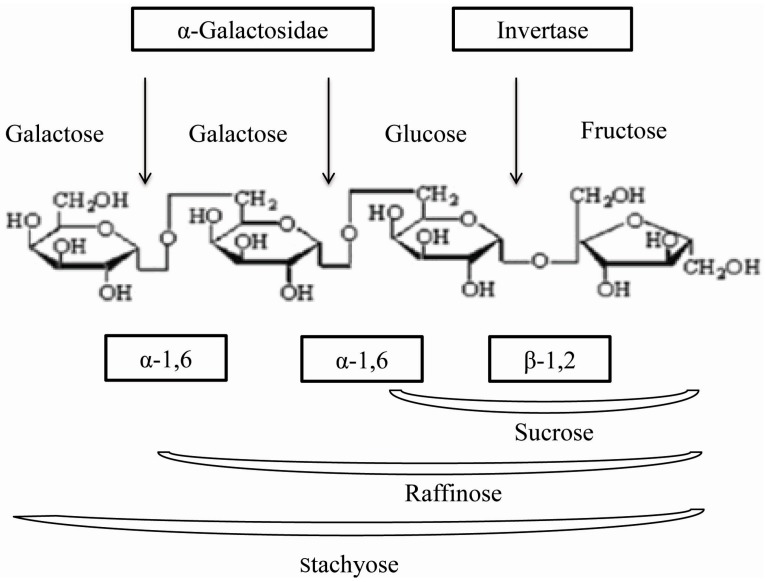
Hydrolysis of oligosaccharides by α-galactosidase and other enzymes.

α-Galactosidases (EC 3.2.1.22) are widely distributed in microorganisms [6,7,8], plants [9] and mammals. These enzymes catalyse the hydrolytic removal of α-1,6-linked α-galactose residues from simple oligosaccharides including melibiose, raffinose, stachyose and polymeric galactomannans (Figure 1) which are employed in the sugar industry to improve sucrose crystallization by hydrolysing raffinose in beet sugar syrups. They are mainly used in the sugar industry, where they improve the crystallization of sucrose by hydrolysing the raffinose in beet sugar syrups [10], also used in the food and feed industries [11,12]. As raffinose, stachyose and some leguminous polysaccharides from seeds of soy-based foods cause intestinal discomfort and flatulence, the hydrolysis of these sugars by α-galactosidase has a positive impact, which would increase the nutritional value of animal feed [13].

In this report, we describe an α-galactosidase isolated from *Termitomyces eurrhizus* which has been identified using the ITS method. This enzyme has good thermostability and acidic stability, and also exihibits high hydrolytic activity towards oligosaccharides.

## 2. Results

The ITS region was estimated to be approximately 800 bp in 1.0% agarose gel. The sequence obtained (gb|KU179194|) was compared with the GenBank (NCBI) (http://blast.ncbi.nlm.nih.gov/) database using the basic local alignment search tool (BLAST) algorithm.

**Figure 2:**
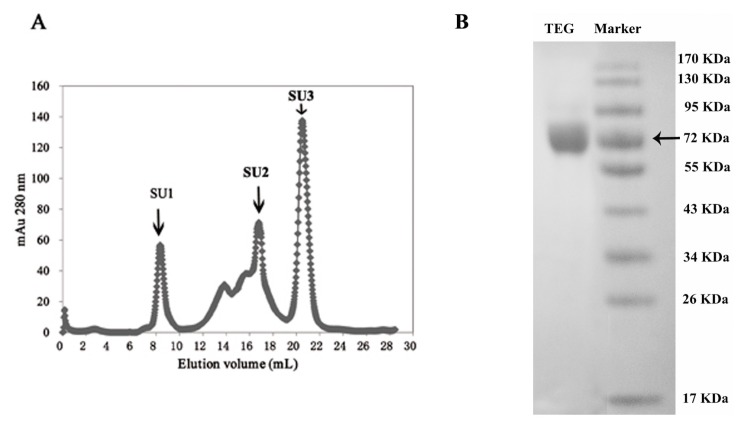
(**A**) Gel-filtration on Superdex 75 HR10/30 column. SU1, SU2 and SU3 stand for the three eluted fractions Fraction SU1 representing purified α-galactosidase; (**B**) Sodium dodecyl sulfate polyacrylamide polyacrylamide gelelectrophoresis (SDS-PAGE) results: **Left** lane: purified α-galactosidase. **Right** lane: molecular mass standards. The molecular mass of SU1 was 72 kDa.

TEG was purified from the fresh fruit bodies of *T. eurrhizus* through the procedures described in Table 1. A 146-fold purification was attained, with a protein recovery rate of 1.4%. The apparent molecular mass of the enzyme was 72 kDa as determined by gel filtration (Figure 2A) and Sodium dodecyl sulfate polyacrylamide polyacrylamide gelelectrophoresis (SDS-PAGE) (Figure 2B), which also demonstrated that TEG was a monomeric protein.

**Table 1:** Summary of purification of TEG.

Chromatographic Fraction	Protein	Total Activity	Special Activity	Yield	Purification
	(mg)	(U) ^a^	(U/mg) ^b^	(%)	Fold ^c^
Crude extract	4384	93,032	21.39	100	1
D2	1120	50,465	45	54.2	2.1
CM2	40.25	26,744	664	28.7	31.1
Q2	6.75	18,418	2728	19.8	127
SU1	0.445	1395	3135	1.4	146

^a^ Total activity: α-galactosidase activity (U/mL) in each step × Volume (mL); ^b^ Specific activity: Total activity/Protein; ^c^ Purification fold: Specific activity after each step/Specific activity after the first step.

SDS-PAGE of the enzyme was conducted and the band corresponding to the purified protein was excised and identification of partial amino acid sequences by electrospray ionisation tandem mass spectrometry (ESI-MS/MS) was carried out. Amino acid sequences of two internal peptide fragments. Peptide 1: LGGAAGTNLEAR and Peptide 2: FSNLDIDSAGR were obtained.

**Figure 3:**
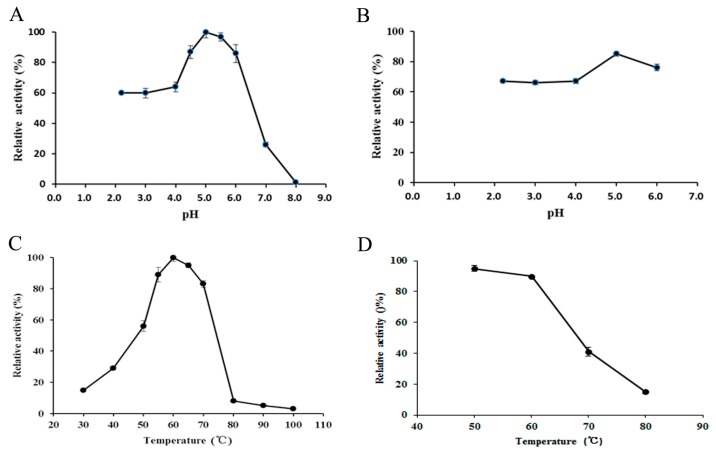
Impact of pH and temperature on α-galactosidase activity. (**A**) Effect of pH on α-galactosidase activity was determined at 50 °C in buffers with pH values from 2.0 to 8.0. The activity at pH 5.0 was defined as 100%; (**B**) pH stability of α-galactosidase activity was demonstrated by determining α-galactosidase activity under standard assay conditions following pre-incubation of the enzyme at room temperature for 2 h in buffers with pH values from 2.0 to 6.0. The activity of an untreated enzyme measured at pH 5.0 was defined as 100%; (**C**) Effect of temperature on α-galactosidase activity was examined at various temperatures in the range 30–100 °C. The activity at 60 °C was considered as 100%; (**D**) Thermostability of α-galactosidase activity was examined following pre-incubation of the enzyme at various temperatures ranging from 50 to 80 °C for 2 h. The activity of enzyme which had not been subjected to heating was defined as 100%. Data represent means ± SD (*n* = 3).

The effect of pH on TEG was determined using pNPGal as the substrate. As shown in Figure 3A,B, TEG displayed the highest activity at pH 5.0, whereas no activity was detectable at pH 8.0. Furthermore, the enzyme was fairly stable over an acidic pH range of 2.0–6.0, retaining more than 65% of its activity after incubation for 120 min.

The optimum temperature and stability on TEG were determined under standard assay conditions. As shown in Figure 3C,D, the enzyme displayed the highest activity at 60 °C. Moreover, the enzyme had good thermal tolerance; no detectable activity was lost after incubation at 60 °C for 120 min.

The effect of various metal ions and chemical modification regents on the activity of TEG have been studied (Table 2). Cd^2+^, Hg^2+^ and Fe^3+^ strongly inhibited the activity of TEG. Mn^2+^, Cu^2+^ and Al^3+^ ions inhibited the activity of TEG only at high concentration (>5 mM). On the other hand, the activity of this enzyme was enhanced by K^+^ and Ca^2+^ ions. Other ions had no effect on the activity of TEG indicating they are not required for catalytic activity.

**Table 2:** Effects of different metal ions on the activity of α-galactosidase (Results represent mean ± SD, *n* = 3).

Metalion	Relative Galactosidase Activity (%)			
Concentration	10 mM	5 mM	2.5 mM	1.25 mM
Fe^2+^	109.77 ± 0.49	113.14 ± 0.25	109.12 ± 2.25	109.77 ± 0.49
K^+^	138.74 ± 2.93	133.07 ± 1.73	128.02 ± 2.91	129.14 ± 0.25
Ca^2+^	148.85 ± 2.14	137.22 ± 1.74	133.25 ± 0.81	123.04 ± 0.64
Cd^2+^	10.07 ± 0.49	9.71 ± 0.36	8.47 ± 0.15	10.38 ± 0.26
Cu^2+^	7.91 ± 0.75	95.03 ± 2.42	107.57 ± 2.22	98.05 ± 0.60
Hg^2+^	11.67 ± 0.35	8.47 ± 0.32	11.02 ± 0.36	14.01 ± 0.87
Mg^2+^	91.31 ± 0.79	89.8 ± 0.55	93.43 ± 2.40	95.68 ± 1.53
Mn^2+^	35.67 ± 2.00	46.91 ± 2.29	63.99 ± 1.84	88.59 ± 2.19
Pb^2+^	116.04 ± 1.02	103.89 ± 0.87	101.99 ± 1.96	97.92 ± 2.07
Zn^2+^	95.11 ± 1.63	116.34 ± 0.66	107.52 ± 2.38	105.53 ± 2.11
Al^3+^	21.49 ± 0.50	61.31 ± 1.78	103.63 ± 1.85	115.48 ± 2.75
Fe^3+^	3.72 ± 0.25	16.99 ± 0.70	20.62 ± 1.82	21.57 ± 1.19

The modification reagents tested with the exception of *N*-bromosuccinimide (NBS), a tryptophan modifying agent [14], did not affect the activity of TEG. Exposure of the enzyme to 0.1 mM NBS for half an hour brought about complete destruction of activity, indicating that tryptophan was paramount to the enzyme activity.

As shown in Table 3, the enzyme was able to hydrolyze pNPGal better than other synthetic substrates such as oNPGal and 4-nitrophenyl β-d-glucuronide. Of the natural substrates tested, the α-galactosidase was most active on the trisaccharide raffinose, followed by the disaccharide melibiose and the tetrasaccharide stachyose. This enzyme was devoid of activity toward locust bean gum and guar gum.

**Table 3:** Hydrolysis of substrates by TEG.

Substrate	Concentration (mM)	Relative Activity ^a^ (%)
*p*NPGal	10	100
*o*NPGal	10	54.38 ± 0.13
4-Nitrophenyl β-d-glucuronide	10	4.95 ± 0.24
Raffinose	50	40.24 ± 0.21
Melibiose	50	20.89 ± 0.20
Stachyose	50	15.11 ± 0.16
Locust bean gum	5%	-
Guar gum	5%	-

^a^ Relative activities were calculated in relation to activity toward pNPGal, which was considered as 100%; - no activity was detected.

**Figure 4:**
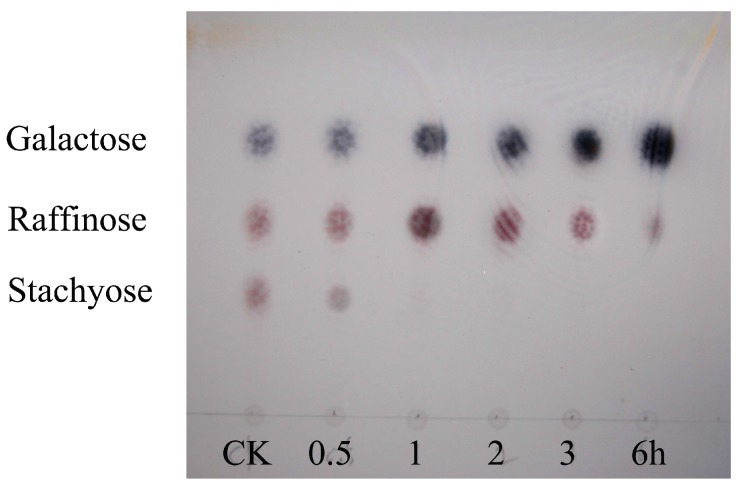
Analysis of products of oligosaccharide hydrolysis by TEG. CK stands for control group (0 min), which indicates oligosaccharides (stachyose, raffinose, galactose) without α-galactosidase treatment.

The hydrolytic activity of TEG on the oligosaccharides was determined by TLC (Figure 4). Raffinose and stachyose, digested for 0.5, 1, 3, 6 h, respectively, showed that the amount of complex oligosaccharides decreased, while the spots of galactose on TLC became more apparent. Stachyose hydrolysis catalyzed by TEG resulted in formation of the products, raffinose, galactose and sucrose in 3 h. Then the accumulated raffinose was completely hydrolyzed to sucrose and galactose in 6 h.

The content of reducing sugar continued to rise with the extension of the duration of incubation, which also indicated that raffinose family oligosaccharides (RFOs) underwent hydrolysis to galactose. At the end of 6 h, the content of reducing sugar had ceased to rise further compared with 12 h (data not shown).

## 3. Discussion

The BLAST search revealed that the partial ITS sequence (accession no. KU179194) showed 93% similarity with that of the *T. eurrhizus* (accession no. KC414235.1), and a 93% similarity with that of the *T. eurrhizus* (accession No. KC414254.1). On the basis of these results, we conclude that *T. eurrhizus* belongs to this species.

Amino acid sequences of two internal peptides within TEG were searched using the NCBI-BLAST database. The results revealed that the sequence of peptide 1 showed 86% similarity with that of *Amycolatopsis mediterranei* (Accession No. YP003765931) and 78% similarity with that of *Trametes versicolor* (Accession No. EIW52338). Peptide 2 showed higher identity to some of the reported glycoside hydrolase family 27 (GH27) protein. The sequence demonstrated low homology to some other fungal α-galactosidase, thus signifying that it is a novel GH 27 α-galactosidase. Microbial α-galactosidases have been classified into GH families 27 and 36, which comprise a superfamily (clan GH-D) [15].

The optimum pH and acidic stability of TEG were somewhat similar to the reported α-galactosidase [16,17,18]. Fungal α-galactosidases are often characterized by a low optimum pH (pH 4.0–6.0), which renders them potentially useful in several applications requiring acidic pH conditions. TEG has no loss of activity after 120 min incubation at 60 °C, which is a useful characteristic of the enzyme to resist high temperatures. Its high thermostabilty is similar to that of α-galactosidases from *Ganoderma lucidum* [19], with an optimum temperature at 70 °C and fully stable to heating at 70 °C for 30 min. However, its thermostability is much higher than that of α-galactosidases from other species like *Penicillium canescens* [20], *Bacillus megaterium* [21], and *Flavobacterium* [22]. TEG was found to be a highly thermostable and acid stable α-galactosidase.

Enzyme activity was indiscernible after incubation with 0.1 mM NBS for half an hour, furnishing evidence that tryptophan plays a crucial role in the enzyme activity. Many studies have demonstrated that NBS can result in complete and partial loss of the enzyme activity by way of attacking tryptophan [23,24,25].

Substrate specificity of TEG was examined by employing oligosaccharides, polysaccharides and synthetic substrates. Generally, α-galactosidases demonstrate superior hydrolytic activity on synthetic substrates (oNPGal, pNPGal) compared to natural substrates (melibiose, raffinose and stachyose) [6,26]. α-Galactosidases can be classified into two groups based on substrate specificity [27]. The first group comprises α-galactosidases possessing activity restricted to oligosaccharides without extensive polymerization, such as melibiose, raffinose, stachyose and short fragments of galacto (gluco) mannans. The second group encompasses α-galactosidases with activity on polymeric substrates. TEG could be grouped under the first category, such as α-galactosidases from *Trichoderma reesei* [28] and *Cicer arietinum* [18]. On the other hand, α-galactosidases from *Clostridium josui* have been shown to act on galactomannans and release galactose [29], such as α-galactosidases from *Bispora* sp. MEY-1 [30].

TLC results and data on reducing sugar content showed that TEG quickly and completely hydrolyzed the raffinose family oligosaccharides. Hence it can be utilized for upgrading the nutritional value of soymilk, a food item often consumed by subjects with lactose intolerance. Earlier investigations on fungal α-galactosidases for elimination of RFOs from soymilk had shortcomings such as low reaction temperatures or extended incubation time owing to unsatisfactory thermostability or lack of thorough enzymatic hydrolysis. [12,31]. α-Galactosidases from *Termitomyces eurrhizus* have good thermostability and completely hydrolyse oligosaccharides.

## 4. Experimental Section

Fresh *Termitomyces eurrhizus* fruiting bodies were obtained from a market in the Yunnan Province of China. DEAE-cellulose and CM-cellulose were obtained from Sigma Chemical CO., St. Louis, MO, USA. Q-Sepharose, SP-Sepharose CL-6B, Superdex 75 HR 10/30 and AKTA Purifier were from GE Healthcare, Uppsala, Sweden. The substrates, pNPGal, guar gum, locust bean gum, melibiose, raffinose, and stachyose were obtained were purchased from Sigma Chemical Company (St. Louis, MO, USA). All other chemicals used were of analytical grade unless otherwise stated.

### 4.1. The Identification on the Genus of Termitomyces

A small piece of the fruiting body was dissolved in 20 μL diluted buffer to extract DNA for about 20 min. An internal transcribed spacer (ITS) region was amplified using the fungal specific primers, ITS1 (5′-TCCGTAGGTGAACCTGCGG-3′) and ITS4 (5′-TCCTCCGCTTATTGATATGC-3′). The amplification was carried out in a 20 μL reaction volume containing template DNA (1 μL), primers (2 μL each), sterilized water (6.6 μL), Hot start II DNA polymerase (0.4 μL) and plant PCR buffer (10 μL). The PCR was performed as follows: initial denaturing step (94 °C, 3 min), (94 °C for 30 s, 55 °C for 30 s, and 72 °C for 2 min), and a final extension step (72 °C, 5 min). PCR products were separated by agarose gel (1%) electrophoresis. PCR products (40 μL) were sequenced using Sanger sequencing by the company Biomed (Beijing, China).

### 4.2. Enzyme Activity Assay

To determine α-galactosidase activity, diluted enzyme solution (50 μL) was incubated at 50 °C for 15 min with the same volume of 10 mM pNPGal in 50 mM sodium acetate buffer (pH 4.5). The reaction was terminated by adding 400 μL 0.5 M Na_2_CO_3_ and the enzyme activity was determined by measuring at 405 nm the amount of *p*-nitrophenol formed. One unit of enzyme activity was defined as the amount of enzyme that liberates 1 μmol of *p*-nitrophenol per minute under the aforementioned conditions [16].

### 4.3. Purification of TEG

*T. eurrhizus* fruiting bodies were blended in saline using a Waring blender. Following extraction overnight at 4 °C, the homogenate was centrifuged (10,000× *g*, 10 min). The resulting supernatant was chromatographed on an anion exchange DEAE-Sephadex column (2.5 × 20 cm) previously equilibrated with 10 mM Tris-HCl buffer (pH 7.2). Following elution of the unadsorbed proteins, fraction D2 eluted with 0.1 M NaCl in the same buffer was found to exhibit enzyme activity. Following dialysis against distilled water, this fraction was chromatographed on a CM-cellulose column (2.5 × 10 cm) previously equilibrated with 10 mM NaOAc-HOAc buffer (pH 3.6). The enzyme activity was eluted using 0.05 M NaCl in the same buffer. The active fraction collected was further resolved by chromatography on an anion exchange Q-Sepharose column (0.5 × 10 cm) previously equilibrated with 10 mM Tris-HCl buffer (pH 7.2). Final purification of the active fraction Q3 was carried out on a Superdex G-75 HR10/30 column by fast protein liquid chromatography using an AKTA Purifier (GE Healthcare, Uppsala, Sweden).

### 4.4. The Molecular Mass of TEG

The isolated α-galactosidase was subjected to SDS-PAGE [32]. Its native molecular mass was determined by gel filtration on an FPLC Superdex 75 HR10/30 column (GE Healthcare). Bovine serum albumin (67 kDa), ovalbumin (43 kDa), ribonuclease A (13 kDa), aprotinin (6.5 kDa) and vitamin B12 (1.4 kDa) were used as molecular weight protein standards in the gel filtration experiment.

### 4.5. Internal Peptide Sequence Analysis of TEG

The protein band excised from the SDS-PAGE gel was dispatched to National Center of Biomedical Analysis (Beijing, China) for partial amino acid sequence analysis. The amino acid sequences of internal peptides of a sample were obtained using high performance liquid chromatography-electrospray tandem mass spectrometry (HPLC-ESI-MS/MS, Thermo Fisher Scientific, Waltham, MA, USA).

### 4.6. Biochemical Properties of TEG

The enzyme activity at different pH values was assayed under standard assay conditions using 0.1 M Na_2_HPO_4_-citric acid buffer (pH range 2.0–8.0). The pH stability was also investigated using the same buffer solutions which were pre-incubated with the enzyme at room temperature for 120 min.

To determine the optimum temperature for the enzyme, enzyme activity was assayed in 0.1 M Na_2_HPO_4_-citric acid buffer (pH 3.0) over the temperature range 4–80 °C for 15 min. To determine the thermal stability of the enzyme, residual enzyme activity was determined under standard conditions (pH 3.0, 50 °C, 15 min) following incubation of the enzyme over the temperature range 50–80 °C for two hours.

The actions of various concentrations of metal ions (Na^+^, K^+^, Ca^2+^, Cd^2+^, Cu^2+^, Hg^+^, Mg^2+^, Mn^2+^, Pb^2+^, Zn^2+^, Al^3+^, and Fe^3+^) and chemical modification reagents, comprising carbodiimide (EDC), diacetyl (DIC), dithiothreitol (DTT), *N*-bromosuccinimide (NBS), and 2, 4, 6-trinitrophenol (TNBS), on TEG activity were examined. Incubation of the enzyme in the presence of these metal ions and chemical modification reagents was carried out at 4 °C for 2 h. Assay of residual enzyme activity was then conducted and the results were compared with the control (enzyme but not reagent was added).

### 4.7. Substrate Specificity of TEG

The enzyme activity against synthetic substrates comprising oNPGal(2-nitrophenyl β-d-galactopyranoside), pNPGal, and 4-nitrophenyl β-d-glucuronide (10 mM) was determined under standard conditions as detailed above in the assay of enzyme activity. The substrate specificity toward raffinose and stachyose was ascertained by determining the reducing sugars formed when the 3,5-dinitrosalicylic acid reagent was employed [33]. α-Galactosidase activity toward melibiose was assayed as mentioned above and the glucose formed was determined using a glucose-oxidase kit (Beijing BHKT Clinical Reagent Co., Ltd., Beijing, China).

### 4.8. Analysis of Hydrolysis Products by TLC

Enzymatic hydrolysis of oligosaccharides belonging to the raffinose family (RFOs) including raffinose and stachyose was investigated by incubating the isolated enzyme (50 μL) with raffinose/stachyose (5 mg/mL) in 50 mM sodium citrate buffer (pH 6.0) at 50 °C for 6 h. Aliquots of the reaction mixture were removed at different time-intervals, subjected to boiling for 5 min to terminate the reaction and the reaction products analyzed by thin-layer chromatography (TLC). The reaction mixtures were applied on a silica gel plate (Merck Silica Gel 60F 254, Darmstadt, Germany), and developed in a propanol:acetic acid:water mixture, 1:1.5:0.1 (by volume). The plates were sprayed with a diphenylamine:phenylamine: acetonum:phosphoric acid mixture (1:1:50:10, *g/v/v/v*), followed by heating in an oven to detect saccharides [34]. Oligosaccharide reduction was also followed by determining the increase of reducing sugars with the 3,5-dinitrosalicylic acid reagent at different time-intervals as previously detailed [33].

## 5. Conclusions

In this study, a new α-galactosidase was purified and characterized from *Termitomyces eurrhizus* which has been identified using the ITS method. The enzyme exhibited good thermostability and stability under acidic conditions. TEG was raffinose- and stachyose-specific and hydrolysis of the oligosaccharides proceeded speedily. These findings signify that TEG possesses tremendous potential in the beet sugar, food and feed industries for elimination of indigestible oligosaccharides from beans.
